# Endotracheal intubation in COVID-19 patients in Brazil: a nationwide survey

**DOI:** 10.5935/0103-507X.20220015-en

**Published:** 2022

**Authors:** Pedro Vitale Mendes, Bruno Adler Maccagnan Pinheiro Besen, Fábio Holanda Lacerda, João Gabriel Rosa Ramos, Leandro Utino Taniguchi

**Affiliations:** 1 Emergency Medicine Discipline, Hospital das Clínicas, Faculdade de Medicina, Universidade de São Paulo - São Paulo (SP), Brazil.; 2 Critical Care Unit, Hospital Otoclinica - Fortaleza (CE), Brazil.; 3 Critical Care Unit, Hospital São Rafael - Salvador (BA), Brazil.; 4 Intensive Care Unit, Hospital Sírio-Libanês - São Paulo (SP), Brazil.

**To the Editor**

Endotracheal intubation is a life-saving procedure in acute respiratory distress syndrome. However, complications such as hypoxia, hypotension and cardiovascular collapse may occur in almost 40% of the procedures in the intensive care unit (ICU).^(1)^ Evidence regarding the best practice of endotracheal intubation in this context is scarce, and most data have been extrapolated from the operating room. In a survey published in *Revista Brasileira de Terapia Intensiva* before the COVID-19 pandemic, neuromuscular blockade was infrequently used in Brazilian ICUs.^(2)^ During the COVID-19 pandemic, the fear of staff contamination may have modified usual practice and contributed to increasing the procedure risk.^(3,4)^ Therefore, in this study, we sought to survey ICU physicians about their practices during airway management in COVID-19 patients. Additionally, we aimed to assess whether the pandemic changed physicians’ strategies regarding the use of neuromuscular blockade and sedation.

A questionnaire was designed using an informal Delphi process among all authors. After ethics approval, we sent an electronic survey to adult ICU physicians. This study was conducted with logistics support from AMIBnet (the Brazilian network of research in ICUs), and the survey was sent to the AMIBnet mailing list. Continuous data are reported as the mean (standard deviation) and median (25th percentile, 75th percentile) as appropriate. Categorical variables are presented as absolute numbers and percentages.

From February 2021 to May 2021, there were 406 respondents from all Brazilian regions, of which 46% were board certified in critical care. The median time from graduation was 10 [6,19] years. Other characteristics of the respondents are provided in table 1. Almost 80% of respondents reported working in an institution with a specific protocol for the intubation of COVID-19 patients. Of the physicians, 41% reported that changes in their usual practice hindered the performance of the procedure and potentially increased the risk of complications (Figure 1). The main differences from previous practice to prevent aerosol dispersion included a direct connection to the mechanical ventilator after endotracheal intubation and the use of devices to occlude the orotracheal tube, which were referred by 56 and 62.5% of the respondents, respectively. The use of personal protective equipment varied among physicians (Table 1). Of the physicians, 91% reported the use of neuromuscular blockade during all or more than 75% of endotracheal intubations, which is much higher than previously reported in our survey.^(2)^ Sedation strategies varied under patient hemodynamic status, and responses did not change with the COVID-19 pandemic.

**Figure 1:**
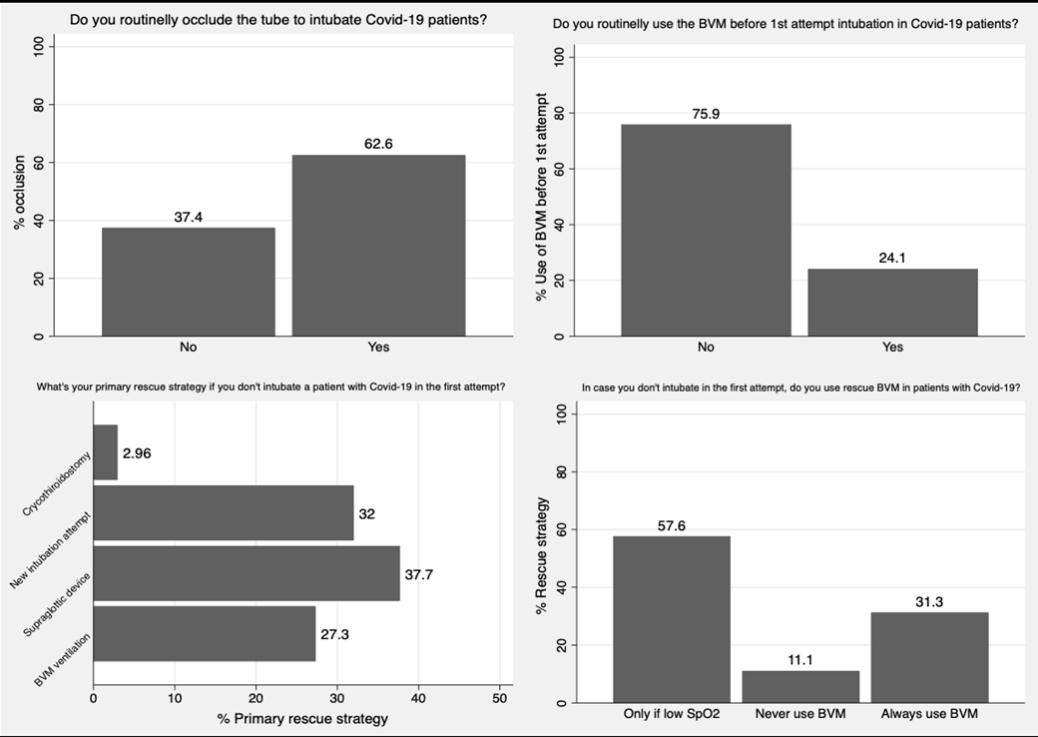
Reported changes in physicians’ usual practice. BVM - bag valve mask; SpO2 - oxygen saturation.

**Table 1:** Respondent characteristics and survey responses regarding endotracheal intubation in COVID-19 patients

Variables	
Male sex	269 (66.3)
Medical residency	
Critical care	179 (44.1)
Internal medicine	224 (55.2)
Surgery	24 (5.9)
Anesthesiology	18 (4.4)
Endotracheal intubation performed monthly	
< 3	87 (21.5)
≥ 3	319 (78.5)
Which PPE do you always use?	
Protective clothing	379 (93.3)
Procedure gloves	396 (97.5)
Protective glasses	251 (61.9)
Surgical face mask	60 (14.8)
N95 respirator mask	398 (98.0)
Disposable cap	345 (85.0)
Face shield	272 (67.0)
Acrylic intubation box	0 (0.0)
PPE - personal protective equipment. Results expressed as n (%).	

We conclude that COVID-19 has changed physicians’ reported practices for endotracheal intubation in Brazilian ICUs.

## References

[1] Simpson GD, Ross MJ, McKeown DW, Ray DC (2012). Tracheal intubation in the critically ill: a multi-centre national study of practice and complications. Br J Anaesth.

[2] Mendes PV, Besen BA, Lacerda FH, Ramos JG, Taniguchi LU (2020). Neuromuscular blockade and airway management during endotracheal intubation in Brazilian intensive care units: a national survey. Rev Bras Ter Intensiva.

[3] Orser BA (2020). Recommendations for endotracheal intubation of COVID-19 patients. Anesth Analg.

[4] Cook TM, El-Boghdadly K, McGuire B, McNarry AF, Patel A, Higgs A (2020). Consensus guidelines for managing the airway in patients with COVID-19: Guidelines from the Difficult Airway Society, the Association of Anaesthetists the Intensive Care Society, the Faculty of Intensive Care Medicine and the Royal College of Anaesthetists. Anaesthesia.

